# A systematic review and meta-analysis on delaying surgery for urothelial carcinoma of bladder and upper tract urothelial carcinoma: Implications for the COVID19 pandemic and beyond

**DOI:** 10.3389/fsurg.2022.879774

**Published:** 2022-10-04

**Authors:** Jeffrey J. Leow, Wei Shen Tan, Wei Phin Tan, Teck Wei Tan, Vinson Wai-Shun Chan, Kari A. O. Tikkinen, Ashish Kamat, Shomik Sengupta, Maxwell V. Meng, Shahrokh Shariat, Morgan Roupret, Karel Decaestecker, Nikhil Vasdev, Yew Lam Chong, Dmitry Enikeev, Gianluca Giannarini, Vincenzo Ficarra, Jeremy Yuen-Chun Teoh, On behalf of the UroSoMe Collaborators

**Affiliations:** ^1^ Department of Urology, Tan Tock Seng Hospital, Singapore, Singapore; ^2^ Lee Kong Chian School of Medicine, Nanyang Technological University, Singapore, Singapore; ^3^ Division of Surgery and Interventional Science, University College London, London, United Kingdom; ^4^ Department of Urology, University College London Hospital, London, United Kingdom; ^5^ Department of Urology, NYU Langone Health, New York City, NY, United States; ^6^ Royal Derby Hospital, University Hospitals of Derby and Burton NHS Foundation Trust, Derby, United Kingdom; ^7^ Leeds Institute of Medical Research, University of Leeds, Leeds, United Kindgom; ^8^ Division of Surgery and Interventional Sciences, University College London, United Kingdom; ^9^ Department of Urology, University of Helsinki and Helsinki University Hospital, Helsinki, Finland; ^10^ Department of Surgery, South Karelian Central Hospital, Lappeenranta, Finland; ^11^ Department of Urology, The University of Texas MD Anderson Cancer Center, Houston, TX, United States; ^12^ Urology Department, Eastern Health, Box Hill, Victoria, Australia; ^13^ Eastern Health Clinical School, Monash University, Box Hill, Victoria, Australia; ^14^ Department of Urology, University of California San Francisco, San Francisco, CA, United States; ^15^ Department of Urology, Comprehensive Cancer Center, Medical University of Vienna, Vienna, Austria; ^16^ Department of Urology, Weill Cornell Medical College, New York, New York, USA; ^17^ Department of Urology, University of Texas Southwestern, Dallas, Texas, USA; ^18^ Department of Urology, Second Faculty of Medicine, Charles University, Prag, Czech Republic; ^19^ Hourani Center for Applied Scientific Research, Al-Ahliyya Amman University, Amman, Jordan; ^20^ Sorbonne University, GRC N 5, Predicitive Onco-uro, AP-HP, Hôpital Pitié-Salpêtriére, Paris, France; ^21^ Department of Urology, AZ Maria Middelares Hospital, Ghent, Belgium; ^22^ Department of Urology, Ghent University Hospital, Ghent, Belgium; ^23^ Department of Human Structure and Repair, Ghent University, Belgium; ^24^ Department of Urology, Hertfordshire and Bedfordshire Urological Cancer Centre, Lister Hospital Stevenage, School of Medicine and Life Sciences, University of Hertfordshire, Hatfield, United Kingdom; ^25^ Institute for Urology and Reproductive Health, Sechenov University, Moscow, Russia; ^26^ Urology Unit, Santa Maria della Misericordia University Hospital, Udine, Italy; ^27^ Department of Human and Pediatric Pathology “Gaetano Barresi”, Urologic Section, University of Messina, Messina, Italy; ^28^ S.H. Ho Urology Centre, Department of Surgery, Prince of Wales Hospital, The Chinese University of Hong Kong, Shatin, Hong Kong SAR, China; ^29^ European Association of Urology – Young Academic Urologists Urothelial Carcinoma Working Group (EAU-YAU), Arnhem, Netherlands

**Keywords:** delay in surgery, delayed treatment, time-to-treatment, urinary bladder neoplasms, ureteral neoplasms, urothelial carcinoma, bladder cancer, bladder carcinoma

## Abstract

**Purpose:**

The COVID-19 pandemic has led to competing strains on hospital resources and healthcare personnel. Patients with newly diagnosed invasive urothelial carcinomas of bladder (UCB) upper tract (UTUC) may experience delays to definitive radical cystectomy (RC) or radical nephro-ureterectomy (RNU) respectively. We evaluate the impact of delaying definitive surgery on survival outcomes for invasive UCB and UTUC.

**Methods:**

We searched for all studies investigating delayed urologic cancer surgery in Medline and Embase up to June 2020. A systematic review and meta-analysis was performed.

**Results:**

We identified a total of 30 studies with 32,591 patients. Across 13 studies (*n* = 12,201), a delay from diagnosis of bladder cancer/TURBT to RC was associated with poorer overall survival (HR 1.25, 95% CI: 1.09–1.45, *p* = 0.002). For patients who underwent neoadjuvant chemotherapy before RC, across the 5 studies (*n* = 4,316 patients), a delay between neoadjuvant chemotherapy and radical cystectomy was not found to be significantly associated with overall survival (pooled HR 1.37, 95% CI: 0.96–1.94, *p* = 0.08). For UTUC, 6 studies (*n* = 4,629) found that delay between diagnosis of UTUC to RNU was associated with poorer overall survival (pooled HR 1.55, 95% CI: 1.19–2.02, *p* = 0.001) and cancer-specific survival (pooled HR of 2.56, 95% CI: 1.50–4.37, *p* = 0.001). Limitations included between-study heterogeneity, particularly in the definitions of delay cut-off periods between diagnosis to surgery.

**Conclusions:**

A delay from diagnosis of UCB or UTUC to definitive RC or RNU was associated with poorer survival outcomes. This was not the case for patients who received neoadjuvant chemotherapy.

## Introduction

Bladder cancer is the 11th most commonly occurring cancer worldwide, with almost 550,000 new cases in 2018 (1, 2). A comprehensive review in 2017 found that bladder cancer ranks 13th in terms of death ranks, with mortality rates decreasing mainly in the most developed countries (3). In comparison, UTUC is much rarer, representing approximately 8.3% of all urothelial carcinoma (4).

At diagnosis, approximately 20% of patients have MIBC (5). One of the factors thought to affect mortality for MIBC is the timing to definitive surgery following diagnosis. The 2020 EAU guidelines cited two studies, with one showing worse clinical outcome and poorer survival in patients who experienced a delay of RC by >3 months while the other showed no survival difference (6, 7). With regards to MIBC patients treated with neoadjuvant chemotherapy, the AUA recommends RC within 6–8 weeks of completion of chemotherapy, unless “medically inadvisable”, while acknowledging that there remains a void of prospective data regarding the optimal timing of RC following NAC (8). Although low grade non-invasive UTUC can be treated endoscopically, RNU remains the treatment of choice for invasive and/or high grade UTUC. The EAU recommends that RNU should not be delayed beyond 12 weeks as this increases the risk of disease progression (9).

This issue of delayed treatment for MIBC and invasive UTUC is especially pertinent in our current ongoing COVID19 pandemic. The severe acute respiratory syndrome coronavirus 2 (SARS-CoV-2) epidemic emerged in December 2019 and has resulted in redistribution of healthcare resources to address the pandemic. This has resulted in cancelation of elective surgeries worldwide (10, 11). Many hospitals have deferred elective and non-cancer surgery, while prioritizing emergency cases and select high-risk oncological cases. To provide expert consensus, the EAU Guidelines Office Rapid Reaction Group recommend that RC should be performed within 3 months from MIBC diagnosis and RNU within 6 weeks of high-risk UTUC diagnosis (12).

The impact of the COVID-19 crisis on elective urological cancer surgery has been significant and disruptive worldwide and is compounded by the concerns of a second or third wave of COVID-19 cases. This invariably will result in the deferment of treatment of localized cancers, which may lead to disease progression and worse survival outcomes. In this study, we performed a systematic review and meta-analysis to evaluate the evidence and association of delayed RC and RNU for patients with MIBC and high-risk UTUC. These data should serve as a framework for decision making regarding timelines of definitive therapy in these disease entities.

## Evidence acquisition

### Protocol registration

Our study methodology was similar to 2 other papers on prostate cancer (13) and kidney cancer (14), whose protocol was registered in the International Prospective Register of Systematic Reviews (PROSPERO) registry (CRD42020190882). We performed this study according to the Preferred Reported Items for Systematic Reviews and Meta-Analyses (PRISMA) guidelines (15). Since most of the included studies were retrospective in nature, we also adhered to guidelines from the “Meta-analysis Of Observational Studies in Epidemiology” (MOOSE) group (16).

### Literature search

We performed a systematic search of PubMed/MEDLINE, Embase, the Cochrane Central Register of Controlled Trials (CENTRAL), and Cochrane Database of Systematic Reviews to identify studies up to June 2020. Different variations of key words and MESH terms for urothelial carcinoma were combined with various combinations of survival outcomes in delaying surgery to identify articles that focused on the issue of delayed surgery. Our complete search strategy is shown in Supplementary Table S1.

#### Objective

The primary objective was to evaluate if delays to RC and RNU would affect the overall survival of patients with MIBC and high-risk UTUC, respectively.

#### Eligibility criteria, manuscript screening, data abstraction, and study quality

We evaluated studies for inclusion and exclusion based on a pre-defined PICOS approach where the population (P), intervention (I), comparator group (C), outcome (O), and study design (S) were considered. This is summarized in Table 1.

**Table 1:** Population, intervention group, comparator group, outcomes and study design (PICO) of studies included in this systematic review and meta-analysis.

Population (P)	Patients diagnosed with invasive urothelial carcinoma of bladder (UCB) or upper urinary tract (UTUC)
Intervention (I)	Radical cystectomy for UCB
	Radical nephro-ureterectomy for UTUC
Comparator group (C)	Delay in surgery
Outcomes (O)	Overall survival
Study design (S)	Retrospective cohort studies
	Prospective cohort studies

### Screening and data extraction

**Figure 1:**
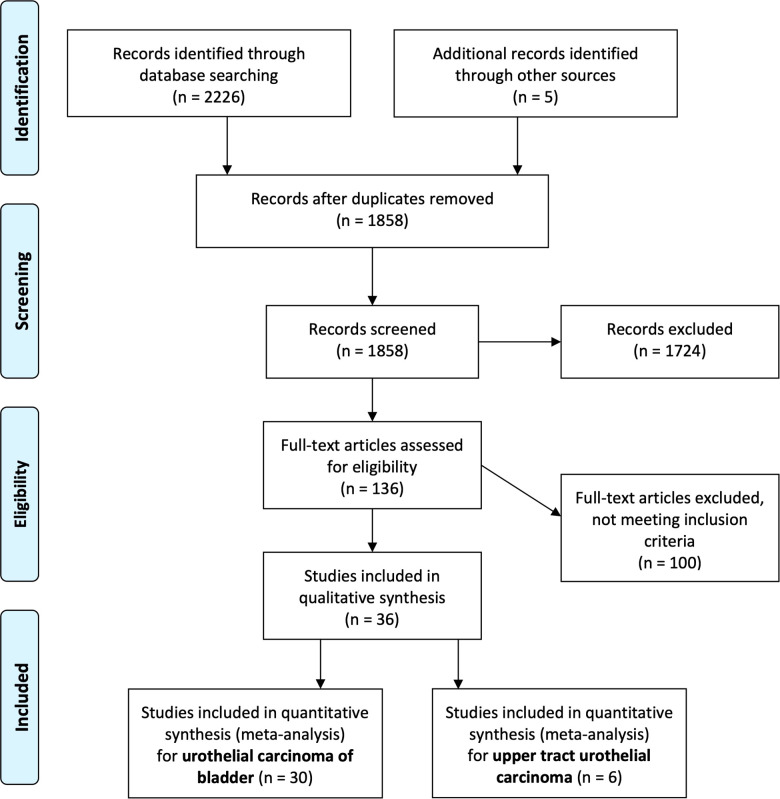
PRISMA flow chart.

Search results were screened by two independent reviewers. Any conflicts were resolved by a third reviewer Finally, eligible articles were identified for full text review (Figure 1). Data extraction was then performed by two authors (JJL, JT) with any discrepancy resolved by a third author (WST). Data on the paper (first author, year, center, country, study design), participant demographics and oncologic characteristics, treatment characteristics, and outcomes, and results were extracted.

### Statistical methods

Descriptive statistics using median and interquartile range were used to summarize demographic and baseline data of eligible patients. Sample size of individual studies, demographic values were calculated based on percentages and summed up to obtain the values used for this cohort. Pooled averages were estimated using fixed and random-effects model when indicated. The *I*^2^ statistic was used to quantify heterogeneity. Statistical analyses were performed using STATA/SE 14.2 (StataCorp, College Station, Texas, USA).

### Risk of bias assessment

We performed risk of bias assessment using the Newcastle-Ottawa Quality Assessment Scale for Cohort Studies (Supplementary Table S2) (17).

## Evidence synthesis

### Search results

Our literature search initially revealed 1,858 articles after removing duplicates. After screening them based on our pre-defined PICOS criteria, we identified 136 articles which were further reviewed in detail and categorized by type of cancer (Figure 1).

### Meta-analysis for bladder cancer studies

We identified a total of 30 studies with 32,591 patients (Table 2). There were varied definitions of delay to RC, with 11 studies identifying the “start point” as “diagnosis of bladder cancer” (18–28), while another 10 used “time of transurethral resection of bladder tumour” (TURBT) (6, 7, 29–36). Five studies evaluated the delay between neoadjuvant chemotherapy and RC (29, 37–40). Four other studies evaluated delay from time of diagnosis prompting BCG therapy to RC (41), time from RC to starting adjuvant chemotherapy (42), time from referral to first treatment (43), and time from first clinic appointment to definitive treatment (radiotherapy or RC) (44).

**Table 2:** Characteristics of included studies evaluating delayed radical cystectomy on survival in bladder cancer and upper tract urothelial carcinoma, based on various definitions of delay: (A) delay between diagnosis of BC and RC; (B) delay between NAC and RC; (C) other definitions of delay.

	Study ID	Year	Journal	Study design	T stage of disease	N stage of disease	Defintion of delay	Delay period cut-off	Median delay	No. of patients who underwent RC	Country of study	Study period	Variable used for multivariable analysis	Key results
**(A) Delay between diagnosis of BC and RC**														
1	Fahmy 2008	2008	Canadian Urological Association Journal	Retrospective	Not available	Not available	1st family practitioner visit to RC	>84 days	93 days	1,633	Canada (Quebec)	1990–2002	Gender, haematuria, year of specialist visit, year of TURBT and radical cystectomy	Multivariate analyses showed that patients with an overall delay of either <25 or >84 days had a 2.1 and 1.4 times increased risk of dying, respectively (*p* < 0.01). A delay of <25 or >84 days between visiting the family physician and RC was associated witha 230% and a 40% increased risk of death from any cause, respectively (95% CI: 1.4–3.9 and 1.1–1.8, respectively).
2	May M 2004	2004	Scandinavian Journal of Urology and Nephrology	Retrospective	cT2-4	N0/N+	Diagnosis of BCa (muscle infiltration) to RC	90 days	55 days	239	Germany		Age, gender, pathologic T and N stage, grade	Patients with a time interval of ≤3 months between diagnosis of muscle invasion and cystectomy had a significantly better progression-free survival rate (55%) than those with a longer time window (34%) (*p* = 0.04). No change in overall survival when RC was delayed, though this relationship was borderline significant (HR = 1.62, 95% CI: 0.99–2.66).
3	Santos 2015	2015	Curr Oncol	Retrospective	Not available	Not available	Diagnosis of BCa to RC (including referral delay)	Patients were considered “indirectly referred” if they made >5 visits to a GP, emergency physician, or other specialist before making a first urology visit. Median delay was 30 days (SD 99).	30 days	1,271	Canada		Age	Patients indirectly referred to a urologist after a first GP visit experienced a 29% increased risk of mortality compared with those directly referred (95% CI: 1.10–1.52).
4	Liedberg 2005	2005	Journal of Urology	Retrospective	cT1-4	N0/N+	Diagnosis of BCa to RC	60 days	49 days	141	Sweden	1990–1997	Age, gender, *de novo* muscle invasive vs. progression to muscle invasion, clinically organ confined T2 disease, type of urinary diversion, preop radiation, referred vs. non-referred	No change in disease-specific survival when there was a delay between diagnosis and RC.
5	Sanchez-Ortis 2003	2003	Journal of Urology	Retrospective	cT2-4	N0/N+	Diagnosis of BCa to RC	<4, 4–6, 7–9, 10–12, 13–16, >16 weeks,	7.9 weeks	290	USA	1987–2000	Pathologic T and N stage, clinical stage	Extravesical disease (P3a or greater) or positive nodes were identified in 84% (16 of 19) of patients when the delay was longer than 12 weeks, compared with 48.2% (82 of 170) in those with a time lag of 12 weeks or less (*p* < 0.01). Similarly 3-year estimated survival was lower (34.9% ± 13.5%) for patients with a surgery delay longer than 12 weeks compared to those with a shorter interval 62.1% ± 4.5% (hazards ratio 2.51, 95% CI 1.30–4.83, *p* = 0.006).
6	Hara 2002	2002	Japanese Journal of Clinical Oncology	Retrospective	cT2-4	N0/N+	Diagnosis of BCa to RC	3 months		50	Japan	1985–2000	-	28 patients who underwent radical cystectomy within 3 months after the primary diagnosis of invasive bladder cancer (group A) and 22 who underwent radical cystectomy more than 3 months after the primary diagnosis (group B). The recurrence-free, cause-specific and overall survival rates in group A were significantly higher than those in group B (*p* < 0.05, *p* < 0.05 and *p* < 0.05, respectively).incidence of vascular involvement in group B was significantly higher than that in group A (*p* < 0.05)
7	Antonelli 2018	2018	Minerva Urologica e Nefrologica	Retrospective	cT1-4	N0/N+	Diagnosis of BCa to RC		76 days	376				Multivariable regression models adjusted for pathological local and lymph nodal stage showed that latency between diagnosis and cystectomy (LDC), continuous or dichotomized at 30/60/90/120/180/240 days was not related to progression-free or overall survival
8	Williams 2017	2017	Urologic Oncology	Retrospective	cT2-4	Not available	Diagnosis of BCa to RC	84 days		9,907	USA (SEER-Medicare)	2001–2011		There was no significant difference in delay to RC according to sex across all clinical stages. Using propensity score matching, women had worse overall (hazard ratio = 1.07; CI: 1.01–1.14; *p* = 0.024), and worse cancer-specific survival (hazard ratio = 1.26; CI: 1.17–1.36, *p* < 0.001) than men. Delay from diagnosis to surgery did not account for this decreased survival among women.
9	Lin-Brande 2019	2019	Urology	Retrospective	cT2-4	Nx/N0/N1	Diagnosis of BCa to RC	<12, ≥12 weeks	Pure urothelial carcinoma: 56 days; Clinical variants: 50 days	363	USA	2003–2014	Age, CCI, LVI, surgical margins, pathologic tumor and lymph node stage	For 363 patients with cT2-T4N0M0 urothelial carcinoma who underwent radical cystectomy without perioperative intravesical and/or systemic therapy from 2003 to 2014, every month in delay was associated with a worse overall survival for variants (HR = 1.36, *p* = 0.003) on multivariable analysis controlling for age, comorbidities, tumor stage, lymph node status, lymphovascular invasion, and surgical margins. At an 8-week delay or longer, those with variant histology had a statistically worse survival (*p* = 0.03).
10	Gore 2009	2009	Cancer	Retrospective	cT2	N0	Diagnosis of BCa to RC	28–56, 56–84, 84–168, ≥168 days		441	USA (SEER-Medicare)	1992–2001		A delay of >12 weeks between diagnosis and RC was associated with a 201% increased risk of all-cause and disease-specific mortality (*p* = 0.003).
11	Lee 2006	2006	Journal of Urology	Retrospective	Not available	Nx/N0/N+	Diagnosis of BCa to RC	93 days	61 days	214	USA	1990–2004		A significant disease specific survival and OS advantage was observed in patients undergoing cystectomy by 93 days or less (3.1 months) compared to greater than 93 days (*p* = 0.05 and 0.02, respectively).
**(B) Delay between TURBT and RC**														
12	Mahmud 2006	2006	Journal of Urology	2	Not available	Not available	TURBT or latest cystoscopy to RC	84 days	33 days	1,592	Canada (Quebec)	1990–2002		After adjusting for calendar year, and patient and provider variables there were no significant differences in survival among the 3 delay categories. However, patients subject to greater than 12 weeks of delay were at 20% greater risk for dying (95% CI 1.0–1.5, *p* = 0.051).
13	Jager 2011	2011	BJU Int	2	Not available	N0/N+	TURBT to RC	120 days	122 days	278	Germany	1989–2006	No of TURBT, tumour extension (bladder confined vs. non-confined), LN metastases, adjuvant therapy, tumour upstaging	Multivariate analysis identified categorized number of TURBs (hazard ratio, HR, 0.14; 95% CI, 0.07–0.44; *p* < 0.001), categorized interval between first TURB and rCx (HR, 3.27; 95% CI, 1.24–8.59; *p *= 0.017), LN status (HR, 0.13; 95% CI, 0.06–0.26; *p* < 0.001) and tumour stage at rCx (HR, 0.49; 95% CI, 0.26–0.92; *p *= 0.03) as independent risk factors for CSS. A delay of >120 days between the first TURBT and RC was associated with significantly worse 5-year cancer-specific survival compared with those with a delay of <120 days (77% vs. 86%).
14	Kulkarni 2009	2009	Journal of Urology	2	Not available	Nx/N0/N+	TURBT to RC	90 days	50 days	2,535	Canada (Ontario)	1992–2004	Socioeconomic status, hospital volume, surgeon volume, surgeon experience, preoperative medical and anaesthetic consultation, preoperative imaging, LIV, perineural invasion, tumour grade, geographic region of residence and year of operation	Unadjusted and adjusted analyses demonstrated that prolonged wait times were significantly associated with a lower overall survival rate. The relative hazard of death with increasing wait times appeared greater for low stage vs. high stage cancers. The cubic splines regression analysis revealed that the risk of death began to increase after 40 days. A delay of >90 days between TURBT and RC was associated with an increased risk of death from all causes compared with those with a delay of 90 days (HR = 1.001, 95% CI: 1.000–1.002). This represents an increased risk of death for each day a patient waits for an RC.
15	Chu 2019	2019	Cancer	2	cT2	N0	TURBT to RC and NAC to RC	TURBT to RC: 84 days; End of NAC to RC: 77 days		1,509	USA (SEER-Medicare)	2004–2012	Age, sex, race, marital status, lymph node status, and comorbidities	In comparison with timely surgery, delays in RC increased overall mortality, regardless of the use of NAC (hazard ratio [HR] without NAC, 1.34; 95% CI, 1.03–1.76; HR after NAC, 1.63; 95% CI, 1.06–2.52).
16	Bruins 2016	2016	Urologic Oncology	2	cT2-4	N0/N+	TURBT to RC	60 days	No NAC: 50 days; NAC: 133 days	1,782	Netherlands (2006–2010)	2006–2010	Age, gender, pathologic T and N stage, referral status, type of treatment hospital (university vs. non-university)	Delayed RC > 3 months was not associated with decreased OS adjusting for confounding variables (hazard ratio = 1.16; 95% CI: 0.91–1.48; *p* = 0.25). Median time from MIBC diagnosis to RC in patients that received neoadjuvant therapy (*n* = 105) was 133 days (interquartile range: 62 days). Adjusting for confounding variables, delayed RC > 3 months was not associated with OS (hazard ratio = 0.90; 95% CI: 0.45–1.82).
17	Kahokehr 2016	2016	ANZ J Surg		cT0-4	N0-2	TURBT to RC	31 days	Mea*n* = 62 days	43	New Zealand	2006–2013		No change in survival when there was a delay between TURBT and RC. Somep patients received neoadjuvant chemotherapy.
18	Nielsen 2007	2007	BJU Int	2	cT2-4	N0/N+	TURBT to RC	90 days	55 days	592	USA	1984–2033		Kaplan-Meier analyses showed no statistical difference in the risk of disease recurrence, disease-specific mortality, or overall mortality between patients who had RC within 3 vs. >3 months after the last TUR (*p *= 0.445, 0.323 and 0.833, respectively) (Figure 1).
19	Ayres 2008	2008	BJU Int	2	Not available	Not available	TURBT to RC	90 days		543	UK	1999–2003		
20	Turk 2018	2018	Tumori	2	cT2-4	Not available	TURBT to RC	3 months		530	Turkey	2005–2016		Patients who underwent delayed RC were compared with patients who were treated with early RC. when both groups were compared for disease-free survival and overall survival, patients of the early-RC group had a greater advantage.
21	Rink 2011	2011	International Journal of Urology	2	cTa-3	pN0-2	TURBT to RC			390				A total of 447 patients who underwent RC between 1996 and 2009 at our institution were considered. Patients were stratified by age (≤70 vs. >70 years). In the elderly, ASA score (*p* < 0.001), delay between transurethral resection of the bladder (TURBT) and RC (*p* = 0.004), and number of perioperative blood transfusions (*p* = 0.002) were significantly higher.
**(C) Delay between NAC and RC**														
22	Alva 2012	2012	Cancer	2	cT2-4	N0/N+	NAC to RC	>12 weeks	From start of NAC: 117 days; From end of NAC: 49 days	153	USA	1990–2007	Univariable	153 patients with MIBC received NAC and underwent radical cystectomy between 1990 and 2007. In multivariate analyses, the timing of cystectomy delivery from the termination of NAC did not significantly alter the risk of survival.
23	Boeri 2019	2019	European Urology Oncology	2	cT2-4	N0-3	End of NAC to RC	>12 weeks	53 days	226	USA	1999–2015	Univariable	The group with time to cystectomy (TTC) >10 weeks had significantly lower OM-free (*p* = 0.003) and CSM-free rates (*p* < 0.001) than the group with TTC ≤10 weeks. TTC was independently associated with higher risk of OM (*p* = 0.027) and CSM (*p* = 0.004) after accounting for age, gender, pathologic extravesical disease, and nodal status.
24	Chu 2019	2019	Cancer	2	cT2	N0	TURBT to RC and NAC to RC	>11 weeks (NAC)		1,509	USA (SEER-Medicare)	2004–2012	Age, sex, race, marital status, lymph node status, and comorbidities	In comparison with timely surgery, delays in RC increased overall mortality, regardless of the use of NAC (hazard ratio [HR] without NAC, 1.34; 95% CI, 1.03–1.76; HR after NAC, 1.63; 95% CI, 1.06–2.52).
25	Park 2016	2016	Journal of Urology	2	cT2-4	N0/N+	Start of NAC to RC	>22 weeks	Diagnosis of BCa to RC: 28 weeks	201	USA	1996–2014	Univariable	Cystectomy performed less than 28 weeks from the diagnosis did not result in significant improvement in overall survival outcomes (HR 0.68, 95% CI 0.28–1.63, *p* = 0.388). Neither the timing of neoadjuvant chemotherapy initiation from diagnosis (median 6 weeks) nor the timing of cystectomy from neoadjuvant chemotherapy initiation (median 22 weeks) was associated with survival.
26	Audenet 2019	2019	Urologic Oncology	2	cT2-4	cN0/pN0/N+	Diagnosis of BCa to start of NAC; Diagnosis of BCa to RC	>6 months	From diagnosis to start of NAC: 39 days; From diagnosis to RC: 112 days	2,227	USA (NCDB)	2004–2014	Univariable	Within the National Cancer Database (2004–2014), we identified 2,227 patients who underwent NAC and RC for cT2-T4aN0M0 urothelial carcinoma of the bladder. We identified delay to NAC ≥8 weeks as a significant cut-off point to predict the risk of upstaging in multivariable analysis (odds ratio: 1.27; 95% CI: 1.02–1.59; *p* = 0.031).
**Other definitions of delay**														
27	Haas 2016	2016	Journal of Urology	2	pT0-4	N0/Nx/N+	Time from diagnosis prompting BCG to RC						Upstaging to cT1 after intravesical therapy, LVI ever, prostatic urethra involvement ever, age	117 patients who underwent radical cystectomy for recurrent nonmuscle invasive bladder cancer at our institution from 1990 to 2012. group 2 = 56 who received at least 1 additional salvage intravesical chemotherapy after bacillus Calmette-Guerin. On multivariate Cox regression analysis delayed cystectomy in group 2 did not convey a significant hazard for all cause mortality after cystectomy (HR 1.08, *p* = 0.808).
28	Booth 2014	2014	Annals of oncology	2	<cT3 and T3-4	N0/Nx/N+	Time from RC to starting adjuvant chemotherapy	1–12 vs. 13–16 weeks	9 weeks	2,944	Canada (Ontario)	1994–2008	Age, socioeconomic status, comorbidity score, pathologic T and N stage, LVI, margin status, comprehensive center status	Ontario Cancer Registry. Of 2,944 patients undergoing cystectomy, 4% (129/2944) and 19% (571/2944) were treated with NACT and ACT, respectively. Time to initiation of ACT (TTAC) was measured from cystectomy.TTAC >12 weeks was associated with inferior OS [hazard ratio (HR) 1.28, 95% CI 1.00–1.62] and CSS (HR 1.30, 95% CI 1.00–1.69).
29	Guilford 1991	1991	BMJ	2	Not available	Not available	Referral to 1st treatment	<27, 27–47, 48–83, ≥84 days	48 days	574	UK	1982	Case severity	
30	Munro 2010	2010	Int J Radiat Oncol	2	Not available	Not available	1st clinic to radiotherapy or RC	<84 vs. ≥84 days		398	UK	1993–1996	Univariable	No change in survival when radiotherapy or RC was delayed

**Figure 2:**
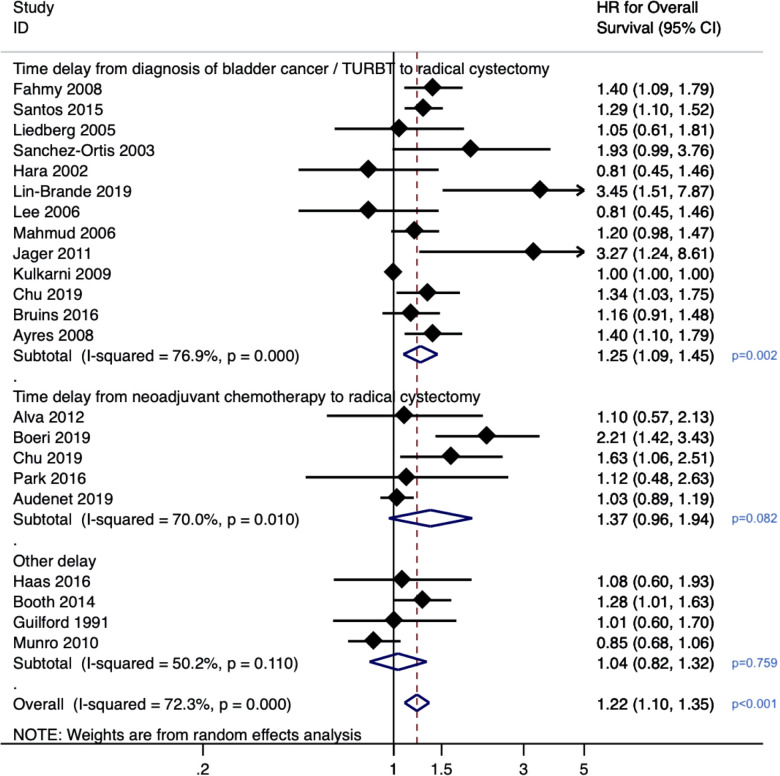
Forrest plot for meta-analysis on effect of delayed radical cystectomy on overall survival in bladder cancer.

Given that the diagnosis of bladder cancer is confirmed upon histology obtained from TURBT, it can be safe to assume that these two “events” are synonymous. Although each study's exact cut-off duration varies from 60 to 90 days, we considered this “delay” the exposure variable for our meta-analysis. Across 13 studies (*n* = 12,201), a delay from diagnosis of bladder cancer/TURBT to RC was associated with poorer overall survival (HR 1.25, 95% CI: 1.09–1.45, *p* = 0.002) (Figure 2). There was substantial heterogeneity with an *I*^2^ value of 76.9% (Cochrane *p*-value <0.001), so a random-effects model was used. Influence analysis showed that the two most influential studies (38, 44) had the greatest effects on the pooled HR if omitted.

For patients who underwent neoadjuvant chemotherapy prior to radical cystectomy, across the five studies (*n* = 4,316 patients), a delay between neoadjuvant chemotherapy and radical cystectomy was not found to be significantly associated with overall survival (pooled HR 1.37, 95% CI: 0.96–1.94, *p* = 0.08). There was substantial heterogeneity with an *I*^2^ value of 70% (Cochrane *p*-value 0.01), so a random-effects model was used. Three studies representing patients treated at Johns Hopkins (40), Michigan (37) (ref) and Mayo (39) reported 3 cycles of neoadjuvant chemotherapy administered and received by patients. The other 2 studies did not have such granular data as they were analyses of the National Cancer Data Base (records only whether patients received single or multi-agent chemotherapy) (38) and SEER-Medicare database (provider billing data utilized to determine receipt and timing chemotherapy) (29).

### Meta-analysis for upper tract urothelial carcinoma studies

**Figure 3:**
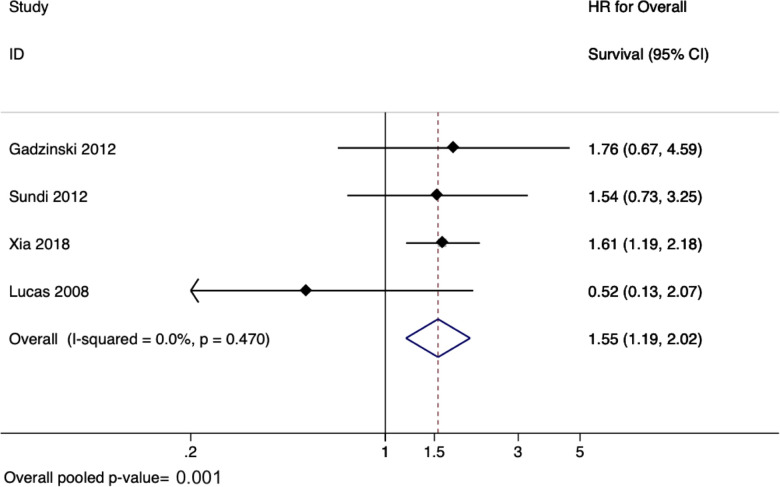
Forrest plot for meta-analysis on effect of delayed radical nephro-ureterectomy on overall survival in upper tract urothelial carcinoma.

**Figure 4:**
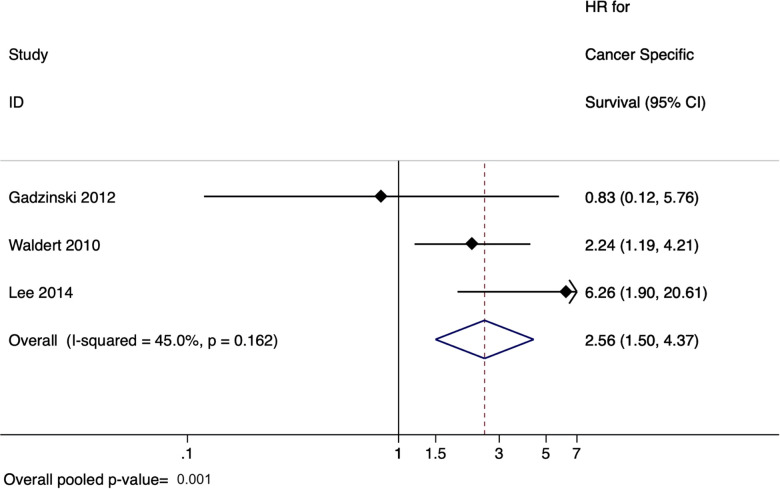
Forrest plot for meta-analysis on effect of delayed radical nephro-ureterectomy on cancer-specific survival in upper tract urothelial carcinoma.

There were six studies evaluating the effect of delay to radical nephroureterectomy on survival for UTUC with a total of 4,629 patients (45–50). When evaluating the delay between diagnosis of UTUC and RNU, the meta-analysis revealed a pooled HR of 1.55 (95% CI: 1.19–2.02, *p* = 0.001) for overall survival (Figure 3) and a pooled HR of 2.56 (95% CI: 1.50–4.37, *p* = 0.001) for cancer-specific survival (Figure 4). There was no evidence of heterogeneity so fixed-effects models were used. Influence analysis showed that Alva et al. (37) had the greatest effect on the result if omitted.

### Discussion

The SARS-CoV-2 epidemic has resulted in the cancelation of elective cancer surgeries worldwide, resulting in delay of cares for patients with invasive urothelial carcinoma. We performed a systematic review and meta-analysis to evaluate the evidence and the effect of delayed RC and RNU for patients with MIBC and high risk UTUC. Our study suggests that for patients who underwent upfront RC, a delay between bladder cancer diagnosis and undergoing definitive RC was associated with significantly poorer overall survival. Similarly, for UTUC, a delay between UTUC diagnosis to RNU was associated with worse overall and cancer-specific survival.

On the contrary, we found that a delay in RC following neoadjuvant chemotherapy did not impact survival outcomes. This finding is particularly pertinent because increasingly more patients with MIBC are receiving neoadjuvant chemotherapy, backed by level one evidence (51). This provides some reassurance to patients who face treatment delays due to chemotherapy related adverse events. Even among a relatively healthy study population in the SWOG-8710 trial, 33% of patients had grade 4 (severe) granulocytopaenia, and 17% had grade 3 (moderate) nausea, vomiting, stomatitis, diarrhoea, or constipation after neoadjuvant chemotherapy (52). However, during the COVID-19 pandemic it is important to acknowledge the theoretical competing risk of succumbing to COVID-19 due to an impaired immune system secondary to chemotherapy (53), particularly among the unvaccinated. This may lead to patients or clinicians electing to avoid peri-operative chemotherapy despite guideline recommendations.

Guidelines and societies have risen to the challenge during the COVID pandemic and came up with suggestions on how to overcome and reduce delay in definitive surgery for urology patients. The Urology Research Network from Italy has strategized how best to reorganize routine urologic practice and recommended how to facilitate the process of rescheduling both surgical and outpatient activities during the COVID-19 pandemic, and in subsequent phases (54). For muscle-invasive bladder cancer, radical cystectomy was categorized in the list of urological surgical procedures strongly recommended to continue during the pandemic, as delay can jeopardise cancer-related outcomes. Caution is advised in case of bowel resection due to high prevalence of high virus load in stool. Preoperative staging is suggested to be simplified to CT chest, abdomen and pelvis, omitting diagnostic ureteroscopy which was optional with weak strength rating in the 2020 EAU guidelines (54, 55). For high-risk UTUC, radical nephro-ureterectomy with template-based lymphadenectomy is also strongly recommended to continue, with preoperative staging simplified to CT urogram and flexible urethrocystoscopy alone, omitting diagnostic ureteroscopy (54, 55). These recommendations are a key referendum for all to resume routine urologic practice and can help as this pandemic evolves with time. Another helpful strategy to improve access for patients with haematuria is to use telehealth services to expedite workup with upper tract imaging and flexible cystoscopy, as described in more detail in a review article highlighting practical ways of how telehealth services can be useful during and after the COVID pandemic (56).

The effect of delays in RC has been investigated previously for MIBC. A recent systematic review (19 studies) and meta-analysis (10 studies) was performed for papers up to August 2019, although we found that there were some methodological errors (e.g., hazard ratio for progression-free survival used in overall survival meta-analysis) (57). Our study has updated the literature search up to June 2020 and includes a total of 30 studies in all, representing the latest available evidence for this topic.

Established dogma would suggest that delays in radical surgery for localised cancer carries the risk of disease progression, resulting in patients missing the opportunity to be cured of their cancer (58). Efforts to minimise treatment delays have led to countries such as the United Kingdom establishing cancer targets for providers to initiate treatment within 31 days from the time decision to treat is established (59). However, it is worth bearing in mind that not all cancer types have the same natural history and prognosis, and in the era of the COVID-19 pandemic, a tailored approached based on cancer disease risk should be adopted in terms of prioritising the urgency of each case. Invasive urothelial carcinoma, in the absence of treatment, progresses quickly. Those who decline treatment with curative intent have a 75% chance of dying from bladder cancer and a 40%–50% chance of doing so within 1 year (60). It may also be possible that delay in surgery could lead to more advanced disease, and could lead to more postoperative complications.

The question of what constitutes an “acceptable” time to treatment delay is often a subject of investigation. A SEER-Medicare analysis of patients with T2 bladder cancer who underwent RC between 1992 and 2001 identified 441 patients. Patients who experienced a delay of 8–12 weeks had a similar mortality risk compared to those who underwent RC within 4–8 weeks of diagnosis. However, patients who experienced a delay of 12–24 weeks had significantly worse mortality (HR 2.0) (27). Similar findings were demonstrated in an analysis of 2,535 patients who underwent RC for bladder cancer in Ontario, Canada between 1992 and 2004 where the hazard ratio of death gradually increased in a step-wise manner with an increase in waiting times. The risk of death exponentially increased when time to treatment was more than 150 days (32).

Causes of treatment delays can be multifactorial. Patients undergoing RC or RNU are often elderly and may have cardiovascular and respiratory comorbidities following years of exposure to cigarette smoking (1, 2). Hence, it is likely this patient cohort requires a multidisciplinary evaluation and a period of “prehabilitation” prior to radical surgery which may result in a delay in time to treatment (61). Patients initially diagnosed in community hospitals may also experience delays when referred to a tertiary unit if referral pathways are not efficient. This is increasingly encountered due to the centralisation of complex cancer surgery. These factors add to the complexities of treatment delays secondary to the COVID-19, where limited healthcare personal, availability of intensive care beds and ventilators, and efforts to minimise staff and patients from contracting COVID-19 significantly impair the ability to provide prompt surgical treatment. As the world moves on from the COVID-19 pandemic, healthcare systems can learn from the gaps exposed and put together comprehensive plans to remedy shortcomings in healthcare inefficiencies, particularly those related to delay in definitive treatment for cancer.

For example, delay in time to treatment following cancer diagnosis only represents part of the treatment pathway. In our current study, we could not account for delays between the interval that a patient experiences symptoms suggestive of possible cancer until the time they seek medical care (62). This may be addressed with bladder health awareness campaigns such as those from the Bladder Cancer Advocacy Network (BCAN), Action Bladder Cancer UK, or World Bladder Cancer Patient Coalition, just to name a few. In addition, delays exist between the time from initial consultation until the completion of investigations, such as staging tests and histopathological confirmation of cancer. Such delays can also influence cancer outcomes and are likely as important to identify and address.

Despite the strengths of our study, it is not devoid of limitations. These include the varying definitions and cut-offs used in individual studies’ analysis of delay, with most studies using a cut-off of 84–93 days. Despite the EAU guideline's recommendations of 12 weeks, numerous studies chose to use different cut-offs to define delays. Additionally, there were insufficient granular data from each study, which limited our ability to perform subgroup meta-regression analysis by T or N stages, for example. Additionally, our meta-analysis was limited to studies published up to June 2020. Finally, there was substantial heterogeneity across different studies, although our meta-analysis attempted to overcome this with random effects models.

## Conclusion

`Our study revealed that a delay between bladder cancer diagnosis and RC was significantly associated with poorer overall survival outcomes, but this was not the case among patients who underwent neoadjuvant chemotherapy prior to RC. Similarly, a delay between UTUC diagnosis and RNU was significantly associated with worse overall and cancer-specific survival. In the COVID-19 era where hospital resources may be limited, we need to continue to provide prompt definitive treatment for our patients with urothelial cancers in order to achieve the best oncologic outcomes for them.

## Data Availability

The original contributions presented in the study are included in the article/**Supplementary Material**, further inquiries can be directed to the corresponding author/s.

## UroSoMe collaborators

We wish to thank the following UroSoMe collaborators who have helped in the initial phase of the project during the COVID-19 pandemic. William Lay Keat Ong, Pratik Gurung, Giacomo Maria Pirola, Luca Orecchia, Matthew Ping Chao Liew, Hsiang-Ying Lee, Yuding Wang, I-Hsuan Alan Chen, Daniele Castellani, Marcelo Langer Wroclawski, Nikhil Mayor, Niranjan J. Sathianathen, Isaac Braga, Zhenbang Liu, Dora Moon.

## References

[1] Bray F Ferlay J Soerjomataram I Siegel RL Torre LA Jemal A. Global cancer statistics 2018: GLOBOCAN estimates of incidence and mortality worldwide for 36 cancers in 185 countries. CA Cancer J Clin. (2018) 68(6):394–424. 10.3322/caac.21492 30207593

[2] Teoh JY Huang J Ko WY Lok V Choi P Ng CF Global trends of bladder cancer incidence and mortality, and their associations with tobacco use and gross domestic product per capita. Eur Urol. (2020) 78(6):893–906. 10.1016/j.eururo.2020.09.006 32972792

[3] Antoni S Ferlay J Soerjomataram I Znaor A Jemal A Bray F. Bladder cancer incidence and mortality: a global overview and recent trends. Eur Urol. (2017) 71(1):96–108. 10.1016/j.eururo.2016.06.010 27370177

[4] Tan WS Feber A Sarpong R Khetrapal P Rodney S Jalil R Who should be investigated for haematuria? Results of a contemporary prospective observational study of 3556 patients. Eur Urol. (2018) 74(1):10–4. 10.1016/j.eururo.2018.03.008 29653885

[5] David KA Mallin K Milowsky MI Ritchey J Carroll PR Nanus DM. Surveillance of urothelial carcinoma: stage and grade migration, 1993-2005 and survival trends, 1993-2000. Cancer. (2009) 115(7):1435–47. 10.1002/cncr.24147 19215030

[6] Bruins HM Aben KK Arends TJ van der Heijden AG Witjes AJ. The effect of the time interval between diagnosis of muscle-invasive bladder cancer and radical cystectomy on staging and survival: a Netherlands cancer registry analysis. Urol Oncol. (2016) 34(4):166.e1–6. 10.1016/j.urolonc.2015.11.006 26705102

[7] Ayres BE Gillatt D McPhail S Cottrell A McGrath J Cottier B A delay in radical cystectomy of >3 months is not associated with a worse clinical outcome. BJU Int. (2008) 102(8):1045. 18840144 10.1111/j.1464-410X.2008.08084_1.x

[8] Chang SS Bochner BH Chou R Dreicer R Kamat AM Lerner SP Treatment of non-metastatic muscle-invasive bladder cancer: AUA/ASCO/ASTRO/SUO guideline. J Urol. (2017) 198(3):552–9. 10.1016/j.juro.2017.04.086 28456635 PMC5626446

[9] Rouprêt M Babjuk M Burger M Capoun O Cohen D Compérat EM European association of urology guidelines on upper urinary tract urothelial carcinoma: 2020 update. Eur Urol. (2020) 79(1):62–79. 10.1016/j.eururo.2020.05.042 32593530

[10] Teoh JY Ong WLK Gonzalez-Padilla D Castellani D Dubin JM Esperto F A global survey on the impact of COVID-19 on urological services. Eur Urol. (2020) 78(2):265–75. 10.1016/j.eururo.2020.05.025 32507625 PMC7248000

[11] Ong WLK Lechmiannandan S Loeb S Teoh JY. Urological services in public hospitals suffered a greater detriment than private hospitals during the battle of COVID-19. Urology. (2020). 10.1016/j.urology.2020.07.010 PMC7367783 32687841

[12] Ribal MJ Cornford P Briganti A Knoll T Gravas S Babjuk M European association of urology guidelines office rapid reaction group: an organisation-wide collaborative effort to adapt the European association of urology guidelines recommendations to the coronavirus disease 2019 era. Eur Urol. (2020). 10.1016/j.eururo.2020.04.056 PMC7183974 32376137

[13] Chan VWS Tan WS Asif A Effects of delayed radical prostatectomy and active surveillance on localised prostate cancer-a systematic review and meta-analysis. Cancers (Basel). (2021) 13(13):3274. 10.3390/cancers13133274 34208888 PMC8268689

[14] Chan VWS Tan WS Leow JJ Delayed surgery for localised and metastatic renal cell carcinoma: a systematic review and meta-analysis for the COVID-19 pandemic. World J Urol. (2021) 39(12):4295–303. 10.1007/s00345-021-03734-1 34031748 PMC8143063

[15] Moher D Liberati A Tetzlaff J Altman DG Group P. Preferred reporting items for systematic reviews and meta-analyses: the PRISMA statement. Br Med J. (2009) 339:b2535. 10.1136/bmj.b2535 19622551 PMC2714657

[16] Stroup DF Berlin JA Morton SC Olkin I Williamson GD Rennie D Meta-analysis of observational studies in epidemiology: a proposal for reporting. Meta-analysis of observational studies in epidemiology (MOOSE) group. J Am Med Assoc. (2000) 283(15):2008–12. 10.1001/jama.283.15.2008 10789670

[17] Wells G Shea B O'Connell D Peterson J Welch V Losos M The Newcastle-ottawa scale (NOS) for assessing the quality of nonrandomised studies in meta-analyses (2020). Available at: http://www.ohri.ca/programs/clinical_epidemiology/oxford.asp (Accessed October 20, 2020).

[18] Fahmy N Kassouf W Jeyaganth S Amin M Mahmud S Steinberg J An analysis of preoperative delays prior to radical cystectomy for bladder cancer in Quebec. Can Urol Assoc J. (2008) 2(2):102–8. 10.5489/cuaj.482 18542741 PMC2422906

[19] May M Nitzke T Helke C Vogler H Hoschke B. Significance of the time period between diagnosis of muscle invasion and radical cystectomy with regard to the prognosis of transitional cell carcinoma of the urothelium in the bladder. Scand J Urol Nephrol. (2004) 38(3):231–5. 10.1080/00365590410029141 15204377

[20] Santos F Dragomir A Kassouf W Franco E Aprikian A. Urologist referral delay and its impact on survival after radical cystectomy for bladder cancer. Curr Oncol. (2015) 22(1):e20–6. 10.3747/co.22.2052 25684993 PMC4324349

[21] Liedberg F Anderson H Mansson W. Treatment delay and prognosis in invasive bladder cancer. J Urol. (2005) 174(5):1777–81; discussion 81. 10.1097/01.ju.0000177521.72678.61 16217282

[22] Sanchez-Ortiz RF Huang WC Mick R Van Arsdalen KN Wein AJ Malkowicz SB. An interval longer than 12 weeks between the diagnosis of muscle invasion and cystectomy is associated with worse outcome in bladder carcinoma. J Urol. (2003) 169(1):110–5; discussion 5. 10.1016/S0022-5347(05)64047-5 12478115

[23] Hara I Miyake H Hara S Gotoh A Okada H Arakawa S Optimal timing of radical cystectomy for patients with invasive transitional cell carcinoma of the bladder. Jpn J Clin Oncol. (2002) 32(1):14–8. 10.1093/jjco/hyf002 11932357

[24] Antonelli A Zamboni S Palumbo C Belotti S Lattarulo M Furlan M Prognostic role of delay before radical cystectomy: retrospective analysis of a single-centre cohort with 376 patients. Minerva Urol Nefrol. (2018) 70(5):494–500. 10.23736/S0393-2249.18.02995-8 29595035

[25] Williams SB Huo J Dafashy TJ Ghaffary CK Baillargeon JG Morales EE Survival differences among patients with bladder cancer according to sex: critical evaluation of radical cystectomy use and delay to treatment. Urol Oncol. (2017) 35(10):602.e1–e9. 10.1016/j.urolonc.2017.05.022 PMC8364004 28647395

[26] Lin-Brande M Pearce SMP Ashrafi AN Nazemi A Burg ML Ghodoussipour S Assessing the impact of time to cystectomy for variant histology of urothelial bladder cancer. Urology. (2019) 133:157–63. 10.1016/j.urology.2019.07.034 31421144

[27] Gore JL Lai J Setodji CM Litwin MS Saigal CS, Urologic Diseases in America P. Mortality increases when radical cystectomy is delayed more than 12 weeks: results from a surveillance, epidemiology, and end results-medicare analysis. Cancer. (2009) 115(5):988–96. 10.1002/cncr.24052 19142878 PMC2654713

[28] Lee CT Madii R Daignault S Dunn RL Zhang Y Montie JE Cystectomy delay more than 3 months from initial bladder cancer diagnosis results in decreased disease specific and overall survival. J Urol. (2006) 175(4):1262–7; discussion 7. 10.1016/S0022-5347(05)00644-0 16515975

[29] Chu AT Holt SK Wright JL Ramos JD Grivas P Yu EY Delays in radical cystectomy for muscle-invasive bladder cancer. Cancer. (2019) 125(12):2011–7. 10.1002/cncr.32048 30840335

[30] Jager W Thomas C Haag S Hampel C Salzer A Thuroff JW Early vs delayed radical cystectomy for ‘high-risk’ carcinoma not invading bladder muscle: delay of cystectomy reduces cancer-specific survival. BJU Int. (2011) 108(8 Pt 2):E284–8. 10.1111/j.1464-410X.2010.09980.x 21244611

[31] Kahokehr A Glasson J Studd R. Surgical waiting time for radical cystectomy: a New Zealand experience. ANZ J Surg. (2016) 86(12):1042–5. 10.1111/ans.13282 26331718

[32] Kulkarni GS Urbach DR Austin PC Fleshner NE Laupacis A. Longer wait times increase overall mortality in patients with bladder cancer. J Urol. (2009) 182(4):1318–24. 10.1016/j.juro.2009.06.041 19683272

[33] Mahmud SM Fong B Fahmy N Tanguay S Aprikian AG. Effect of preoperative delay on survival in patients with bladder cancer undergoing cystectomy in Quebec: a population based study. J Urol. (2006) 175(1):78–83; discussion. 10.1016/S0022-5347(05)00070-4 16406875

[34] Nielsen ME Palapattu GS Karakiewicz PI Lotan Y Bastian PJ Lerner SP A delay in radical cystectomy of >3 months is not associated with a worse clinical outcome. BJU Int. (2007) 100(5):1015–20. 10.1111/j.1464-410X.2007.07132.x 17784888

[35] Rink M Dahlem R Kluth L Minner S Ahyai SA Eichelberg C Older patients suffer from adverse histopathological features after radical cystectomy. Int J Urol. (2011) 18(8):576–84. 10.1111/j.1442-2042.2011.02794.x 21699582

[36] Turk H Un S Cinkaya A Kodaz H Parvizi M Zorlu F. Effect of delayed radical cystectomy for invasive bladder tumors on lymph node positivity, cancer-specific survival and total survival. Tumori. (2018) 104(6):434–7. 10.5301/tj.5000626 28665471

[37] Alva AS Tallman CT He C Hussain MH Hafez K Montie JE Efficient delivery of radical cystectomy after neoadjuvant chemotherapy for muscle-invasive bladder cancer: a multidisciplinary approach. Cancer. (2012) 118(1):44–53. 10.1002/cncr.26240 21598245

[38] Audenet F Sfakianos JP Waingankar N Ruel NH Galsky MD Yuh BE A delay ≥8 weeks to neoadjuvant chemotherapy before radical cystectomy increases the risk of upstaging. Urol Oncol. (2019) 37(2):116–22. 10.1016/j.urolonc.2018.11.011 30509868

[39] Boeri L Soligo M Frank I Boorjian SA Thompson RH Tollefson M Delaying radical cystectomy after neoadjuvant chemotherapy for muscle-invasive bladder cancer is associated with adverse survival outcomes. Eur Urol Oncol. (2019) 2(4):390–6. 10.1016/j.euo.2018.09.004 31277775

[40] Park JC Gandhi NM Carducci MA Eisenberger MA Baras AS Netto GJ A retrospective analysis of the effect on survival of time from diagnosis to neoadjuvant chemotherapy to cystectomy for muscle invasive bladder cancer. J Urol. (2016) 195(4 Pt 1):880–5. 10.1016/j.juro.2015.11.024 26598426

[41] Haas CR Barlow LJ Badalato GM DeCastro GJ Benson MC McKiernan JM. The timing of radical cystectomy for bacillus calmette-guerin failure: comparison of outcomes and risk factors for prognosis. J Urol. (2016) 195(6):1704–9. 10.1016/j.juro.2016.01.087 26807928

[42] Booth CM Siemens DR Peng Y Tannock IF Mackillop WJ. Delivery of perioperative chemotherapy for bladder cancer in routine clinical practice. Ann Oncol. (2014) 25(9):1783–8. 10.1093/annonc/mdu204 24915872

[43] Gulliford MC Petruckevitch A Burney PG. Survival with bladder cancer, evaluation of delay in treatment, type of surgeon, and modality of treatment. Br Med J. (1991) 303(6800):437–40. 10.1136/bmj.303.6800.437 1912834 PMC1670565

[44] Munro NP Sundaram SK Weston PM Fairley L Harrison SC Forman D A 10-year retrospective review of a nonrandomized cohort of 458 patients undergoing radical radiotherapy or cystectomy in yorkshire, UK. Int J Radiat Oncol Biol Phys. (2010) 77(1):119–24. 10.1016/j.ijrobp.2009.04.050 19665319

[45] Lucas SM Svatek RS Olgin G Arriaga Y Kabbani W Sagalowsky AI Conservative management in selected patients with upper tract urothelial carcinoma compares favourably with early radical surgery. BJU Int. (2008) 102(2):172–6. 10.1111/j.1464-410X.2008.07535.x 18341624

[46] Waldert M Karakiewicz PI Raman JD Remzi M Isbarn H Lotan Y A delay in radical nephroureterectomy can lead to upstaging. BJU Int. (2010) 105(6):812–7. 10.1111/j.464-410X.2009.08821.x 19732052

[47] Gadzinski AJ Roberts WW Faerber GJ Wolf JS Jr. Long-term outcomes of immediate versus delayed nephroureterectomy for upper tract urothelial carcinoma. J Endourol. (2012) 26(5):566–73. 10.1089/end.2011.0220 21879886

[48] Sundi D Svatek RS Margulis V Wood CG Matin SF Dinney CP Upper tract urothelial carcinoma: impact of time to surgery. Urol Oncol. (2012) 30(3):266–72. 10.1016/j.urolonc.2010.04.002 20869888 PMC3034106

[49] Lee JN Kwon SY Choi GS Kim HT Kim TH Kwon TG Impact of surgical wait time on oncologic outcomes in upper urinary tract urothelial carcinoma. J Surg Oncol. (2014) 110(4):468–75. 10.1002/jso.23589 25059848

[50] Xia L Taylor BL Pulido JE Guzzo TJ. Impact of surgical waiting time on survival in patients with upper tract urothelial carcinoma: a national cancer database study. Urol Oncol. (2018) 36(1):10.e5–e22. 10.1016/j.urolonc.2017.09.013 29031419

[51] Vale C, Collaboration ABCM. Neoadjuvant chemotherapy in invasive bladder cancer: a systematic review and meta-analysis. Lancet. (2003) 361(9373):1927–34. 10.1016/S0140-6736(03)13580-5 12801735

[52] Grossman HB Natale RB Tangen CM Speights VO Vogelzang NJ Trump DL Neoadjuvant chemotherapy plus cystectomy compared with cystectomy alone for locally advanced bladder cancer. N Engl J Med. (2003) 349(9):859–66. 10.1056/NEJMoa022148 12944571

[53] Jee J Foote MB Lumish M Stonestrom AJ Wills B Narendra V Chemotherapy and COVID-19 outcomes in patients with cancer. J Clin Oncol. (2020). 32795225 10.1200/JCO.20.01307 PMC7571792

[54] Ficarra V Novara G Abrate A Bartoletti R Crestani A De Nunzio C Urology practice during the COVID-19 pandemic. Minerva Urol Nefrol. (2020) 72(3):369–75. 10.23736/S0393-2249.20.03846-1 32202401

[55] Simonato A Giannarini G Abrate A Bartoletti R Crestani A De Nunzio C Clinical pathways for urology patients during the COVID-19 pandemic. Minerva Urol Nefrol. (2020) 72(3):376–83. 10.23736/S0393-2249.20.03861-8 32225135

[56] Novara G Checcucci E Crestani A Abrate A Esperto F Pavan N Telehealth in urology: a systematic review of the literature. How much can telemedicine be useful during and after the COVID-19 pandemic? Eur Urol. (2020) 78(6):786–811. 10.1016/j.eururo.2020.06.025 32616405 PMC7301090

[57] Russell B Liedberg F Khan MS Nair R Thurairaja R Malde S A systematic review and meta-analysis of delay in radical cystectomy and the effect on survival in bladder cancer patients. Eur Urol Oncol. (2019). 31668714 10.1016/j.euo.2019.09.008

[58] Richards M. The national awareness and early diagnosis initiative in England: assembling the evidence. Br J Cancer. (2009) 101(S2):S1. 10.1038/sj.bjc.6605382 19956152 PMC2790704

[59] England N. Achieving world-class cancer outcomes: a strategy for England 2015-2020. London, UK 2015.

[60] de Vere White R Dall'Era M. RE: quality indicators for bladder cancer services: a collaborative review. Eur Urol. (2021) 79(5):700. 10.1016/j.eururo.2020.11.031 33288273

[61] Psutka SP Barocas DA Catto JWF Gore JL Lee CT Morgan TM Staging the host: personalizing risk assessment for radical cystectomy patients. Eur Urol Oncol. (2018) 1(4):292–304. 10.1016/j.euo.2018.05.010 31100250

[62] Ngo B Perera M Papa N Bolton D Sengupta S. Factors affecting the timeliness and adequacy of haematuria assessment in bladder cancer: a systematic review. BJU Int. (2017) 119(Suppl 5):10–8. 10.1111/bju.13821 28544294

