# Supplementary material for: A systematic review and meta-analysis on delaying surgery for urothelial carcinoma of bladder and upper tract urothelial carcinoma: Implications for the COVID19 pandemic and beyond
Source: Front Surg. 2022 Oct 4;9:879774. doi: 10.3389/fsurg.2022.879774 (PMC9577485; doi:10.3389/fsurg.2022.879774)
Supplement: Supplementary file 1 [file Table1.docx]

**Supplementary Table 1**. Search strategy

Database: OVID Medline Epub Ahead of Print, In-Process & Other Non-Indexed Citations, Ovid MEDLINE(R) Daily and Ovid MEDLINE(R) 1946 to Present, Embase <1974 to 2020 March 27>, EBM Reviews - Cochrane Central Register of Controlled Trials <February 2020>, EBM Reviews - Cochrane Database of Systematic Reviews <2005 to March 19, 2020>

Search Strategy:

--------------------------------------------------------------------------------

4 exp *Urinary Bladder Neoplasms/ (100722)

5 ((bladder or vesical) adj2 (cancer* or carcinoma* or malignan* or tumor* or tumour* or neoplas* or papilloma)).tw,kw. (116769)

6 exp *Carcinoma, Transitional Cell/ (31281)

7 (transitional cell adj2 (cancer* or carcinoma* or malignan* or tumor* or tumour* or neoplas*)).tw,kw. (24335)

8 ((NMIBC or MIBC) adj2 bladder).tw,kw. (5480)

20 ((urology or urological) and (cancer* or carcinoma* or malignan* or tumor* or tumour* or neoplas*)).ti. (4432)

21 (((upper urinary tract or urothelial or urothelium) and (cancer* or carcinoma* or malignan* or tumor* or tumour* or neoplas*)) or UTUC).ti. (22448)

22 or/1-21 (817664)

25 exp Cystectomy/ (37071)

26 cystectom*.tw,kw. (39490)

27 ((en-bloc adj2 resect*) or TURBT or TUR or TURB or ERBT or EBTUR or EBTURBT or RERBT or ETURBT).tw,kw. (25408)

28 exp transurethral resection/ (23262)

29 ((transurethral or trans-urethral) and resect* and bladder).tw,kw. (18556)

43 or/23-42 (636619)

44 22 and 43 (223059)

45 (delayed or postpone* or post-pone* or deferred or deferring or temporize* or prolong* or (chanage* adj2 date) or put off or hold back or suspended or timing or active surveillance).tw,kw. (1946748)

46 ((watchful adj3 waiting) or (watch adj3 wait)).tw,kw. (10213)

47 or/45-46 (1955047)

48 44 and 47 (16617)

49 exp Disease-Free Survival/ or exp Survival Analysis/ or exp Survival/ or exp Survival Rate/ or exp survival/ (1565249)

50 (surviv* or "DFS" or "DSS").tw. (2866070)

51 (death* or died or mortality or mortalities or deceased or dead* or fatal* or lethal*).tw. (4387512)

52 exp Mortality/ (1437737)

53 or/49-52 (6874740)

54 48 and 53 (9027)

55 (exp animals/ or exp animal/ or exp nonhuman/ or exp animal experiment/ or animal model/ or animal tissue/ or non human/ or (rat or rats or mice or mouse or swine or porcine or murine or sheep or lambs or pigs or piglets or rabbit or rabbits or cat or cats or dog or dogs or cattle or bovine or monkey or monkeys or trout or marmoset$1).ti.) not (humans/ or human/ or human experiment/ or (human* or men or women or patients or subjects).tw.) (10315014)

56 54 not 55 (8968)

57 conference abstract.pt. or Congresses as Topic/ or Conference Review.pt. or "Journal: Conference Abstract".pt. (4030060)

58 56 not 57 (6673)

59 limit 58 to english language [Limit not valid in CDSR; records were retained] (5951)

60 note/ or editorial/ or letter/ or Comment/ or news/ or (note or editorial or letter or Comment or news).pt. (4596452)

61 59 not 60 (5793)

62 remove duplicates from 61 (3422)

***************************
