# Supplementary material for: A systematic review and meta-analysis on delaying surgery for urothelial carcinoma of bladder and upper tract urothelial carcinoma: Implications for the COVID19 pandemic and beyond
Source: Front Surg. 2022 Oct 4;9:879774. doi: 10.3389/fsurg.2022.879774 (PMC9577485; doi:10.3389/fsurg.2022.879774)
Supplement: Supplementary file 2 [file Table2.docx]

**Supplementary Table 2.** Risk of bias assessment using Newcastle-Ottawa Quality Assessment Scale for Cohort Studies

|  |  |  |  |  | | | | | | | | | | | | |  |  |
| --- | --- | --- | --- | --- | --- | --- | --- | --- | --- | --- | --- | --- | --- | --- | --- | --- | --- | --- |
|  | **Study ID** | **Year** | **Journal** | **Selection 1** | **Selection 2** | | **Selection 3** | | **Selection 4** | **Comparability A** | | **Comparability B** | **Outcomes 1** | | **Outcomes 2** | | **Outcomes 3** | **Scores** |
|  | **Delay between diagnosis of BC and RC** | | |  |  | |  | |  |  | |  |  | |  | |  |  |
| 1 | Fahmy 2008 | 2008 | Canadian Urological Association Journal | * | * | | * | | * | * | | * | * | | * | | - | 8 |
| 2 | May M 2004 | 2004 | Scandinavian Journal of Urology and Nephrology | * | * | | * | | * | * | | * | * | | * | | - | 8 |
| 3 | Santos 2015 | 2015 | Curr Oncol | * | * | | * | | * | - | | - | * | | * | | - | 6 |
| 4 | Liedberg 2005 | 2005 | Journal of Urology | * | * | | * | | * | - | | - | * | | * | | - | 6 |
| 5 | Sanchez-Ortis 2003 | 2003 | Journal of Urology | * | * | | * | | * | * | | - | * | | * | | - | 7 |
| 6 | Hara 2002 | 2002 | Japanese Journal of Clinical Oncology | * | * | | * | | * | - | | - | * | | * | | - | 6 |
| 7 | Antonelli 2018 | 2018 | Minerva Urologica e Nefrologica |  |  | |  | |  |  | |  |  | |  | |  |  |
| 8 | Williams 2017 | 2017 | Urologic Oncology | *EXCLUDED STUDY AS DID NOT REPORT OUTCOME OF INTEREST* | | | | | | | | | | | | | | |
| 9 | Lin-Brande 2019 | 2019 | Urology | * | * | | * | | * | * | | * | * | | * | | - | 8 |
| 10 | Gore 2009 | 2009 | Cancer | * | * | | * | | * | - | | - | * | | * | | - | 6 |
| 11 | Lee 2006 | 2006 | Journal of Urology | * | * | | * | | * | - | | - | * | | * | | - | 6 |
|  | **Delay between TURBT and RC** | | |  |  | |  | |  |  | |  |  | |  | |  |  |
| 12 | Mahmud 2006 | 2006 | Journal of Urology | * | * | | * | | * | - | | - | * | | * | | - | 6 |
| 13 | Jager 2011 | 2011 | BJU Int | * | * | | * | | * | * | | * | * | | * | | - | 8 |
| 14 | Kulkarni 2009 | 2009 | Journal of Urology | * | * | | * | | * | * | | * | * | | * | | - | 8 |
| 15 | Chu 2019 | 2019 | Cancer | * | * | | * | | * | * | | * | * | | * | | - | 8 |
| 16 | Bruins 2016 | 2016 | Urologic Oncology | * | * | | * | | * | * | | * | * | | * | | - | 8 |
| 17 | Kahokehr 2016 | 2016 | ANZ J Surg | *EXCLUDE STUDY AS DID NOT REPORT OUTCOME OF INTEREST* | | | | | | | | | | | | | | |
| 18 | Nielsen 2007 | 2007 | BJU Int | *EXCLUDE STUDY AS DID NOT REPORT OUTCOME OF INTEREST* | | | | | | | | | | | | | | |
| 19 | Ayres 2008 | 2008 | BJU Int | * | * | | - | | - | - | | - | * | | - | | - | 3 |
| 20 | Turk 2018 | 2018 | Tumori | *EXCLUDE STUDY AS DID NOT REPORT OUTCOME OF INTEREST* | | | | | | | | | | | | | | |
| 21 | Rink 2011 | 2011 | International Journal of Urology | *EXCLUDE STUDY AS DID NOT REPORT OUTCOME OF INTEREST* | | | | | | | | | | | | | | |
|  | **Delay between NAC and RC** | | |  |  | |  | |  |  | |  |  | |  | |  |  |
| 22 | Alva 2012 | 2012 | Cancer | * | * | | * | | * | * | | - | * | | * | | - | 6 |
| 23 | Boeri 2019 | 2019 | European Urology Oncology | * | * | | * | | * | * | | - | * | | * | | - | 8 |
| 24 | Chu 2019 | 2019 | Cancer | * | * | | * | | * | * | | - | * | | * | | - | 8 |
| 25 | Park 2016 | 2016 | Journal of Urology | * | * | | * | | * | - | | - | * | | * | | - | 6 |
| 26 | Audenet 2019 | 2019 | Urologic Oncology | * | * | | * | | * | - | | - | * | | * | | - | 6 |
|  | **Other definitions of delay** | | |  |  | |  | |  |  | |  |  | |  | |  |  |
| 27 | Haas 2016 | 2016 | Journal of Urology | * | * | | * | | * | - | | * | * | | - | | - | 6 |
| 28 | Booth 2014 | 2014 | Annals of oncology | * | * | | * | | * | * | | * | * | | * | | - | 8 |
| 29 | Guilford 1991 | 1991 | BMJ | * | * | | * | | * | - | | - | * | | * | | - | 6 |
| 30 | Munro 2010 | 2010 | Int J Radiat Oncol | * | * | | * | | * | - | | - | * | | * | | - | 6 |
| **NEWCASTLE - OTTAWA QUALITY ASSESSMENT SCALE COHORT STUDIES** | | | | | |  | |  | | |  | | |  | |  | | |
| ***Reference: Wells, G. A, Shea, B., O'Connel, D. et al. The Newcastle-Ottawa scale (NOS) for assessing the quailty of nonrandomised studies in meta-analyses. http://www ohri ca/programs/clinical_epidemiology/oxford htm 2009 Feb 1*** | | | | | | | | | | | | | | | | | | |
